# Dispersion of *Legionella* bacteria in atmosphere: A practical source location estimation method

**DOI:** 10.1371/journal.pone.0224144

**Published:** 2019-11-25

**Authors:** Steven Dyke, Iain Barrass, Kevin Pollock, Ian M. Hall

**Affiliations:** 1 Emergency Response Department Science and Technology (ERD S&T), Public Health England, Porton Down, Wiltshire, United Kingdom, SP4 0JG; 2 Health Protection Scotland, Glasgow, United Kingdom; 3 School of Health and Life Sciences, Glasgow Caledonian University, Glasgow, United Kingdom; Universidade de Coimbra, PORTUGAL

## Abstract

Legionnaires’ disease, a form of pneumonia which can be fatal, is transmitted via the inhalation of water droplets containing *Legionella* bacteria. These droplets can be dispersed in the atmosphere several kilometers from their source. The most common such sources are contaminated water within cooling towers and other air-conditioning systems but other sources such as ornamental fountains and spa pools have also caused outbreaks of the disease in the past. There is an obvious need to locate and eliminate any such sources as quickly as possible. Here a maximum likelihood model estimating the source of an outbreak from case location data has been developed and implemented. Unlike previous models, the average dose exposure sub-model is formulated using a atmospheric dispersion model. How the uncertainty in inferred parameters can be estimated is discussed. The model is applied to the 2012 Edinburgh Legionnaires’ disease outbreak.

## Introduction

The number of notified cases of Legionnaires’ disease, a potentially fatal atypical form of pneumonia, within the EU/EEA has increased each year since 2013 reaching 9,238 cases in 2017. Although most of these were sporadic cases in 2017, 28 outbreaks in 9 different countries were reported (ECDC [1]). Legionnaires’ disease is primarily caused by the inhalation of bacteria from the genus *Legionella*, most commonly *L. pneumophila*, contained within aerosolised water from a contaminated source (Naik and Dabrera [2]). (Outbreaks caused by other transmission routes such as by *L. longbeachae* within compost (Currie and Beattie [3]) are not considered in this paper). Examples of such sources have included cooling towers and other air-conditioning units (Addiss et al. [4], Bennett et al. [5], Brown et al. [6], Brown et al. [7], Castellani Pastoris et al. [8], García-Fulgueiras et al. [9], Jansà et al. [10], Keramarou et al. [11], Kirrage et al. [12], Nguyen et al. [13]), an asphalt paving machine (Coscollá et al. [14]), an industrial air scrubber (Nygård et al. [15]), spa pools (Coetzee et al. [16]) and decorative fountains (Correia et al. [17]). Outbreaks can last a number of weeks (Egan et al. [18]), over which time the wind, which can have a significant effect on the dispersion of the contaminated aerosol, will have changed considerably and there is no certain knowledge of where cases may have acquired their infections. The dose response for humans is poorly understood, though some studies have considered this for guinea pigs, monkeys, mice and rats (Armstrong and Haas [19], Prasad et al. [20]).

Analytical tools to analyse outbreaks have been published. These range from simple data visualisation (that is mapping of case locations with contextual data layers) [14], potential source scoring system [12], attack ratio analysis [6, 10, 13, 15] to more advanced statistical models and analysis ([6, 9, 17], Martinez-Beneito et al. [21] cited in Bull et al. [22]). Other approaches include comparison of case positions and modelled dispersion plume [13, 15], identifying the ‘release window’ by deconvolving the epidemic curve and incubation period [18] and cluster detection to provide linkage in space and time between cases (Sansom et al. [23]). Reverse epidemiology as described in Legrand et al. [24] would be applicable only if there is reason to be confident that the release was over a short duration.

These methods assume *a priori* knowledge of potential sources in an area. Both Hancock et al. [25] and van Leuken et al. [26] use maximum likelihood approaches to overcome this, as is done in this paper. This also allows potential sources to be effectively compared which is not possible with many of the simpler but pragmatic and effective earlier methods. Hancock et al. base their likelihood on the movement of individuals using probabilities derived from data contained within a large database and assume, without attempting to give any mechanistic justification, that only individuals within a hexagon containing the source can be infected and that the infection rate within this hexagon is constant. Although van Leuken et al. apply their method to Q fever it is equally applicable to Legionnaires’ disease. Within their binomial based likelihood they assume that the concentration of the causative agent decays exponentially with distance from the source. In this paper a more detailed use of atmospheric dispersion theory is used in an attempt to obtain a method with a greater mechanistic justification.

To simplify the problem, censoring and reporting biases during the evolution of an outbreak are neglected and only the spatial pattern of cases is considered. This means that it is assumed that no spatial bias is present in the data, such as might arise if there was a dose dependency on the incubation period of the disease. Whilst such a dose dependency is evident for other bacterial infections, evidence for temporal dose dependency for legionella is limited. Prasad et al. [20] used *L.longbeachae* A/J mice data due to being unable to find more suitable data to fit their time-dose response model. Given a home, work or visit location for each case it may be surmised that this is the location of infection and so a model is required to identify, given all the currently identified case locations, where a potential source may be. If a model of dissemination from a source can be developed then, using some fitting process, one can obtain the point in space that best explains the data given the model, some uncertainty around this point and a framework for comparing other known *a priori* potential sources in the area.

To develop a more mechanistic model for source identification an appeal to concepts from atmospheric dispersion modelling is made. A packet of air containing aerosolised legionella bacteria is subject to conservation of mass, that is the packet is not destroyed but simply changes volume. The bacteria will initially be suspended in water droplets which will dry out over time depending on atmospheric conditions. This process, or the atmospheric conditions directly, may kill or reduce the viability of the bacteria (Hambleton et al. [27]) but such processes are the subject of ongoing research (for example Pourchez et al. [28] which considers viability after inhalation) and are neglected here for the sake of simplicity. The packet is considered to be passive, that is it has no reactive impact on the atmospheric gases surrounding it, and the molecular diffusion coefficient for an atmospheric situation is assumed to be small. However, local to the packet the flow field is likely to be turbulent (Stockie [29]). Eddies of varying size are formed and act on the packet in different ways depending on the size of the eddy in relation to the packet. These eddies are formed by apparently insignificant perturbations to the flow field.

The Materials and methods section of this paper outlines the derived model and the procedures used to estimate the parameters and their uncertainty. The following section describes the results of applying these to data from the 2012 Edinburgh Legionnaires’ disease outbreak which had 61 confirmed or probable cases, including 4 deaths (Irons et al. [30]). The paper concludes with a discussion on possible further improvements to the model.

## Materials and methods

For this model it is assumed that the geographical area of interest can be partitioned into a number of regions or cells each with known population and a representative point, such as a geographical or population centroid. In the results presented in this paper the model is applied to an outbreak within Great Britain and GB postcodes are taken to define these spatial cells but other choices are available such as administrative units. The cells should be non-overlapping but cover the entire area under consideration. Furthermore, it is assumed that for each case of the disease a list of possible infection locations, specified as a cell, is available and that it is possible to order the elements of each such list based on the chances of each location being the true infection location.

It is assumed that the number of cases within each cell is given by a Poisson distribution informed by dose within that cell. The mean of this distribution is calculated from diffusion-advection equations with constant wind. The diffusion coefficients are taken to be constant over a cell and given by the Briggs formulation, (see for example Hanna [31]). This leads to, (see S1 Appendix), the expected number of cases in a cell being taken as

$$\begin{array}{c} \lambda =\alpha \frac{{\left(1+br\right)}^{c}{\left(1+b_{z}r\right)}^{c_{z}}}{r^{2}}\exp(-\frac{H^{2}{\left(1+b_{z}r\right)}^{2c_{z}}}{2a_{z}^{2}r^{2}})P\;\left(\text{for}\;r>0\right) \end{array}$$ (1)

where

*α* is a super-parameter common to all cells,
*b*, *c*, *a*_*z*_, *b*_*z*_, *c*_*z*_ are Briggs parameters common to all cells,
*H* is the height above ground of the source,
*P* is the population of the cell

and

*r* is the horizontal distance of the cell from the source.

The Briggs parameters are taken to be from a super-set of values varying with whether the release is over open-country or an urban area and on the Pasquill stability class. Six Pasquill classes, (A to F), are considered but for urban environments two pairs share parameters, so effectively there are only four types in this case. It is assumed that whether the release is open-country or urban is known and therefore the Briggs parameters can be considered to be a known function of a categorical parameter *p* representing the (unknown) Pasquill stability class.

The appearance of the height of the source, *H*, only in the exponential factor of the expected number of cases in a cell as given by Eq (1) means that the likelihood varies little with *H* when *H* or *r* is large (as *c*_*z*_ ≤ 1, see Table A.1 of S1 Appendix). This leads to extremely unrealistic high values of *H* when attempts are made to fit *H*, therefore it is assumed that the height of the source is known. A value of 2m was used as this is representative of a source at head-height, which was the case in the Barrow-in-Furness outbreak [5]. It is believed that the model is not very sensitive to the value chosen, (see Results).

Thus the model has four unknown parameters, *X*_*s*_, *Y*_*s*_, *p* and *α*. Denoting the total number of cells within the considered geographical region by *N* and the number of cases within the *i*-th one by *n*_*i*_ ≥ 0, the log-likelihood of the parameters is

$$\begin{array}{c} l\left(X_{s},Y_{s},p,\alpha \right)=-\sum_{i=1}^{N}\lambda_{i}+\sum_{i=1}^{N}n_{i}\log\lambda_{i}-\sum_{i=1}^{N}\log\left(n_{i}!\right) \end{array}$$ (2)

where λ_*i*_ is the expected number of cases in the *i*-th cell as given by Eq (1). This is used to estimate the parameters via a maximum likelihood estimator, (MLE), as follows.

### Parameter estimation

From Eq (1) it can be seen that λ = *α*λ^(1)^ where λ^(1)^ is the value of λ if the super-parameter *α* has value 1. It is easy to show that the MLE of *α* is given by

$$\begin{array}{c} \hat{\alpha }=\frac{\sum_{i=1}^{N}n_{i}}{\sum_{i=1}^{N}{\hat{\lambda }}_{i}^{\left(1\right)}} \end{array}$$ (3)

where ${\hat{\lambda }}_{i}^{\left(1\right)}$ is the value of $\lambda_{i}^{\left(1\right)}$ evaluated at the MLE values of *X*_*s*_, *Y*_*s*_ and *p*. Thus the problem is reduced from four to three parameters. The search for the MLE values of these is reduced to a finite manageable one by assuming that the source is located at the representative location of one of a reasonably small number of cells. Within the results presented in this paper, the set of possible source postcodes, (cells being taken as postcodes), is taken as those within the postal district, (as given by the first part of the postcode), having the largest prevalence of the disease of all postal districts.

As both source and cases are effectively assumed to occur at the representative point, the likelihood of cases within the source cell is undefined due to zero not being in the range of *r* for which Eq (1) is valid. This is overcome by taking the location of these cases to be as far from the source location as possible whilst remaining in the cell.

### Parameter uncertainty

A common way of assessing the uncertainty of MLEs is the use of confidence regions based on twice the likelihood ratio being approximately *χ*^2^-distributed. However, this relies on being able to expand the log-likelihood as a Taylor series about the MLEs. This is not possible here as one of the parameters, namely the stability class *p*, is categorical. In order to overcome this, the inference problem of finding estimates for the four parameters *X*_*s*_, *Y*_*s*_, *α* and *p*, is recast as one in model selection with the likelihood associated with each of the stability classes being considered a different model. Model selection will be based on the Akaike information criterion (AIC) for each model. The AIC of a model with *K* parameters is given by

$$\begin{array}{c} AIC=2K-2l(\hat{\theta }) \end{array}$$

where $l(\hat{\theta })$ is the maximum log-likelihood of the model. The best model is taken as the model with the smallest AIC value. As here all models have the same number of parameters, namely 3, the selected model is the one with the greatest maximum log-likelihood and therefore the recast problem will generate exactly the same parameter values as the original approach. (This would remain true if the small sample corrected AIC or the Bayesian information criterion was used instead of the AIC). However, the new approach allows the strength of the evidence for each model (stability class) to be assessed, as described by Burnham and Anderson [32], either through the differences in the AICs directly or using them to calculate Akaike weights which can be viewed as the probability that each model is the best one. The scaling criterion value for each model is taken as

$$\begin{array}{c} \Delta =AIC-AIC_{min} \end{array}$$

where *AIC*_*min*_ is the AIC of the selected model. The Akaike weight of each model is then equal to exp(− Δ/2) but scaled so that the sum over all the models is one. Note that as each model has the same number of parameters, these weights are equivalent to scaled maximum likelihoods.

Burnham and Anderson advocate a model averaging approach to inference but here the stability class will be taken as having the value giving the best model and the uncertainty in the other parameters will be considered. As all three remaining parameters are continuous, it will be assumed that a 95% confidence region is approximated by

$$\begin{array}{c} \left\{\left(X_{s},Y_{s},\alpha \right)|l\left({\hat{X}}_{s},{\hat{Y}}_{s},\hat{\alpha }\right)-l\left(X_{s},Y_{s},\alpha \right)<c\right\} \end{array}$$

where *c* is half of the 95% percentile of the *χ*^2^-distribution with 3 degrees of freedom and ^ denotes the MLE of a parameter. The parameter *p* has been dropped from the argument list of *l* as it is now fixed. The projection of this onto the (*X*_*s*_, *Y*_*s*_) plane is given by

$$\begin{array}{c} \{\left(X_{s},Y_{s}\right)|\max_{\alpha }l\left(X_{s},Y_{s},\alpha \right)>l\left({\hat{X}}_{s},{\hat{Y}}_{s},\hat{\alpha }\right)-c\}. \end{array}$$

For computational ease this was approximated by

$$\begin{array}{c} \{\left(X_{s},Y_{s}\right)|\max_{\alpha ,p}l\left(X_{s},Y_{s},p,\alpha \right)>l\left({\hat{X}}_{s},{\hat{Y}}_{s},\hat{p},\hat{\alpha }\right)-c\}. \end{array}$$

In order to quantify the uncertainty in the super-parameter *α* it will be assumed that *X*_*s*_, *Y*_*s*_ and *p* all take their fitted MLE values and a 95% Wald confidence interval will be calculated as

$$\begin{array}{c} (\hat{\alpha }-\frac{1.96}{\sqrt{I(\hat{\alpha })}},\hat{\alpha }+\frac{1.96}{\sqrt{I(\hat{\alpha })}}), \end{array}$$

where *I*(*α*) is the Fisher information which is given by

$$\begin{array}{c} I\left(\alpha \right)=-E(\frac{\partial^{2}l}{\partial \alpha^{2}}). \end{array}$$

From Eq (1) is can be seen that

$$\begin{array}{c} \frac{\partial \lambda }{\partial \alpha }=\frac{\lambda }{\alpha }. \end{array}$$

Using this it follows from Eq (2) that

$$\begin{array}{c} \frac{\partial^{2}l}{\partial \alpha^{2}}=-\frac{M}{\alpha^{2}} \end{array}$$

where *M* is the predicted total number of cases. The expected value of *M* is given by

$$\begin{array}{c} E\left(M\right)=\sum_{i=1}^{N}\lambda_{i}. \end{array}$$

Using the same working that lead to Eq (3) gives that at $\alpha =\hat{\alpha }$ this is equal to the observed total number of cases, *M*_*o*_ say. Thus the desired value of the Fisher information is

$$\begin{array}{c} I\left(\hat{\alpha }\right)=\frac{M_{o}}{{\hat{\alpha }}^{2}} \end{array}$$

and the wanted Wald confidence interval is given by

$$\begin{array}{c} (\hat{\alpha }(1-\frac{1.96}{\sqrt{M_{o}}}),\hat{\alpha }(1+\frac{1.96}{\sqrt{M_{o}}})). \end{array}$$ (4)

## Results

The model was implemented in the statistical programming environment R, with GB postcodes acting as the cells of the model.

The model was run using case data from the Legionnaires’ disease outbreak that occurred in Edinburgh, UK in 2012 (NHS Lothian [33]). At the time of this outbreak it was investigated by an Incident Management Team (IMT) with members from NHS Lothian, Health Protection Scotland, City of Edinburgh Council, Health and Safety Executive (HSE) and Scottish Haemophilus, Legionella, Meningococcus and Pneumococcus Reference Laboratory. They conducted a systematic search for possible sources, including but not restricted to cooling towers, which must by law be registered. This led to a number of measures being taken by environmental health and HSE. Further details can by found in [33] and McCormick et al. [34].

The rest of this section describes the application of the model to the data by the authors in an attempt to validate the model. It did not form part of the IMT’s investigation. Four case locations were removed from the data before applying the model because these locations were too distant from the other locations to be realistic infection locations. Three were outside of the Edinburgh postal area. It is believed that it is very uncommon for Legionnaires’ disease infections to occur more that 10km from the source and that this would require a high velocity source such as an industrial air-scrubber [15].

Two sets of possible infection locations were considered. The first, referred to as Sample 1, consisted of the home location of the cases where a valid postcode for this was known and the population within the postcode was also known. The second, referred to as Sample 2, used work locations instead of home ones if they were available.

From the data the postal district with the highest disease prevalence per head of population was found to be EH11 and therefore the search for the predicted source location was confined to this district.

**Fig 1:**
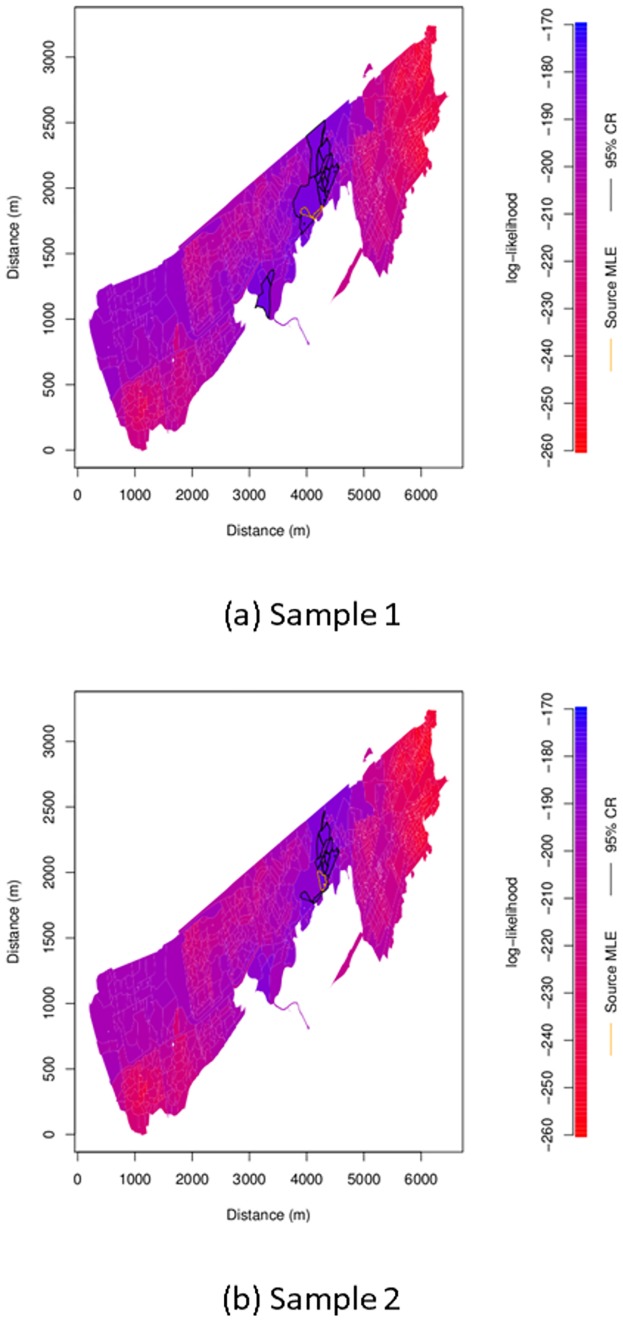
Maximum log-likelihood heat map. Heat map of maximum log-likelihood within search district EH11 with inferred source location postcode estimate outlined in orange. Postcodes within the approximate 95% confidence region calculated as described under Parameter Uncertainty are outlined in black.

Heat maps of the log-likelihood for each sample are shown in Fig 1. The maps are overall similar. The two source location estimates, which are outlined in orange, are adjacent postcodes. Postcodes lying in the approximate 95% confidence regions calculated using the method given under Parameter Uncertainty are outlined in black. The Sample 2 confidence region can be seen to be largely contained within the Sample 1 one with 12 of its 14 postcodes being amongst the 17 postcodes making up the Sample 1 confidence region.

**Fig 2:**
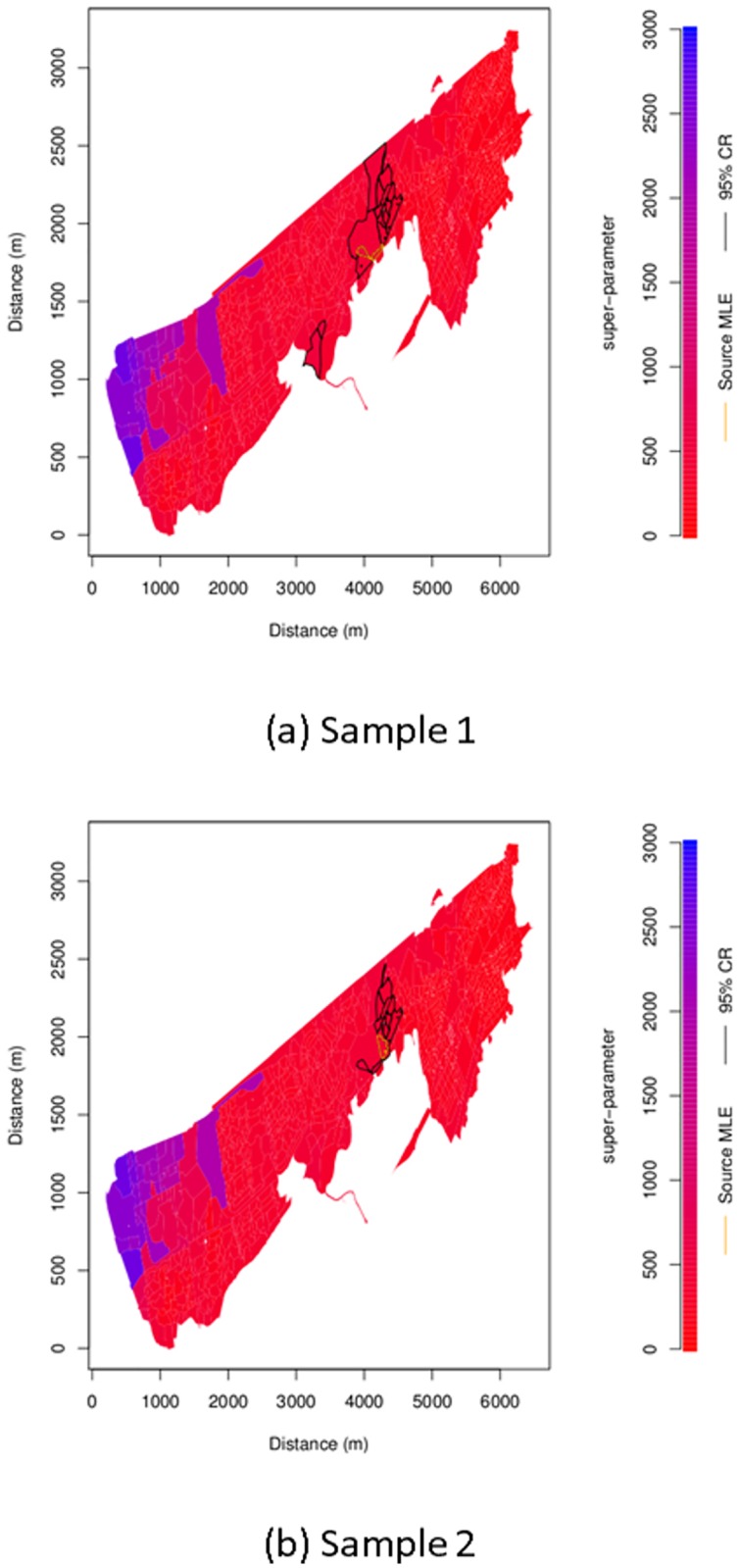
Super-parameter *α* heat map. Heat map of super-parameter *α* at maximum log-likelihood within search district EH11 with inferred source location postcode estimate outlined in orange. Postcodes within the approximate 95% confidence region calculated as described under Parameter Uncertainty are outlined in black.

Fig 2 shows heat plots of the value of the super-parameter *α* when the parameters are set to their maximum log-likelihood values for postcodes in the search district of EH11. The two plots are similar. The value of super-parameter is reasonably constant over the search area except near the North-West extreme where there were no cases.

**Fig 3:**
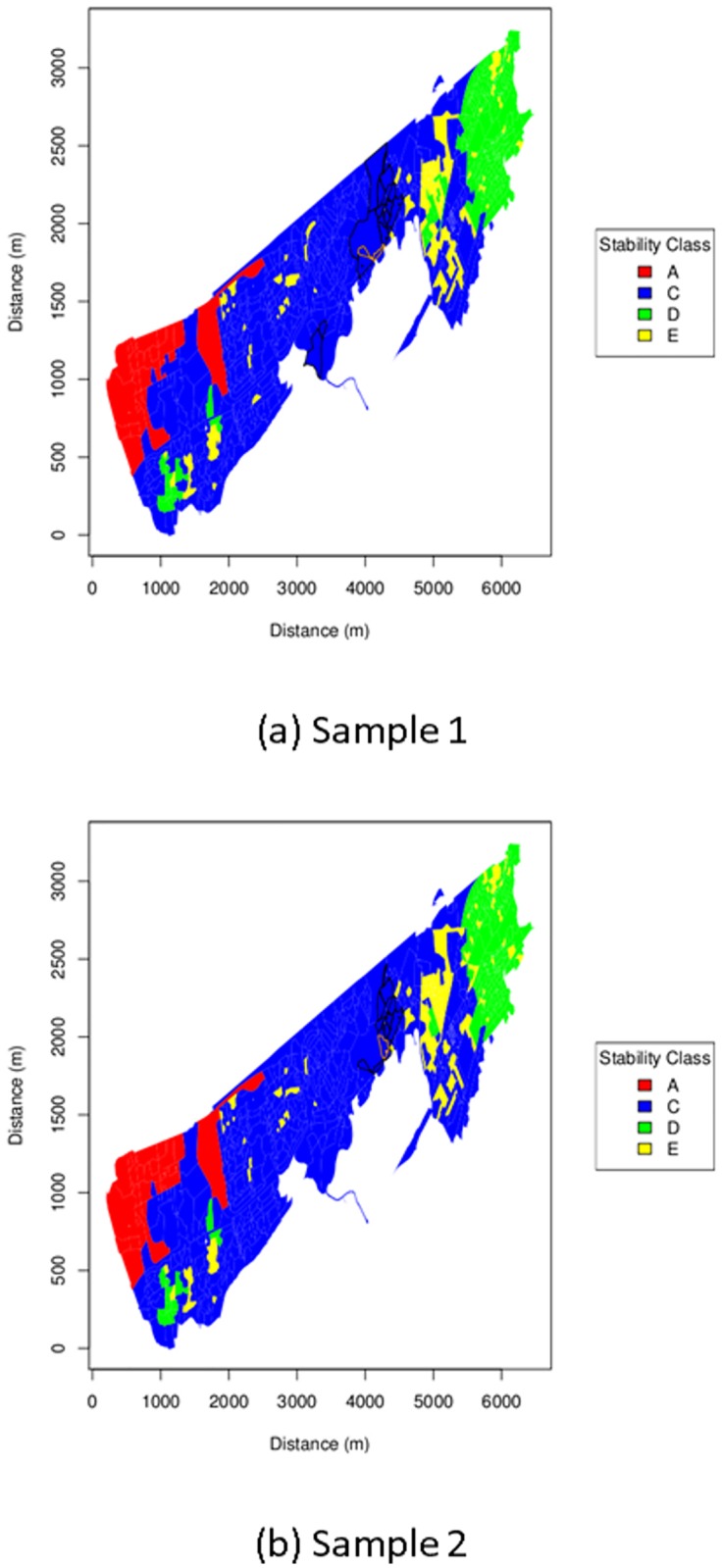
Stability class map. Map with postcodes coloured to represent the stability class at maximum log-likelihood within search district EH11 with inferred source location postcode estimate outlined in orange. Postcodes within the approximate 95% confidence region calculated as described under Parameter Uncertainty are outlined in black.

The stability classes giving the maximum log-likelihood for each possible source position considered within EH11 are shown in Fig 3. Once again the Sample 1 and Sample 2 plots are similar. In both the most frequent class by far is C followed by D. The confidence regions are entirely class C. Class A occurs only in the North-West of the search area, far from the confidence regions.

The numerical values of the maximum likelihood values over each stability class, the scaling criterion values and the Akaike weights are shown in Table 1. From this it can be seen that both samples suggest the same fit ordering of the stability classes of C, A-B, E-F, D. Using the rules of thumb quoted by Burnham and Anderson, the evidence for the A-B class is not substantial, (which would require Δ ≤ 2), and there is effectively no evidence in favour of class E-F, (as Δ > 10). The 3 source postcodes given by the 8 model/sample pairs are all in close proximity with circle passing through their centroids having a radius of less than 370m.

**Table 1:** Stability class model selection statistics and parameter estimates (with dummy postcodes for confidentiality reasons).

(**a**) Sample 1					
Stability Class	Max log-likelihood	Δ	Akaike Weight	Source Postcode	*α*
A-B	-181.8497	4.587496	0.091211693	EH11 A	734.6122
C	-179.5560	0.000000	0.904091777	EH11 A	456.6739
D	-186.1620	13.211962	0.001222563	EH11 C	221.9015
E-F	-185.1176	11.123268	0.003473967	EH11 C	256.2893
(**b**) Sample 2					
Stability Class	Max log-likelihood	Δ	Akaike Weight	Source Postcode	*α*
A-B	-182.9996	6.312058	0.040546631	EH11 B	583.2370
C	-179.8435	0.000000	0.951920825	EH11 B	387.4137
D	-186.4495	13.211916	0.001287270	EH11 B	241.4565
E-F	-184.8702	10.053314	0.006245274	EH11 B	281.6705

As for both samples *M*_*o*_ = 29, using data from Table 1 in Eq (4) gives that the super-parameter *α* confidence interval for Sample 1 is (290.5, 622.9)m^2^ and for Sample 2 is (246.4, 528.4)m^2^.

As part of the official investigation of the outbreak, two types of spatial modelling were used, sophisticated large code dispersion modelling and attack rate analysis [33]. The former produced two possible infection regions within which the source may be located. EH11 A is on the boundary of the first of these near the south west corner and EH11B is stretches between the two. EH11 A is approximately 400 to 500m to the south east of the high attack rate area. Therefore the model presented here is in reasonably good agreement with the methods used at the time of the outbreak.

In order to consider the effect of the choice of 2m for the fixed value of the source height, the calculations were repeated except with the source height set to 5m and 10m. The predicted source postcode is unchanged for both heights from those obtained using a height of 2m, as is the predicted stability class. This is also true for the second favoured stability class with the values of Δ also remaining similar. For the lesser favoured stability classes, *E* − *F* is always a better fit than *D*, but possible new source postcodes are introduced, say EH11 D, EH11 E and EH11 F, where EH11 E is adjacent to EH11 B, C, D and F. The greastest distance between the centroids of any of the postcodes EH11 A to F is approximately 560m between EH11 A and EH11 E.

## Discussion

The results given here were found by first estimating the parameters assuming that all cases were infected at their home location, then repeating the process assuming that all cases were infected at their work location. It is unlikely that all infections occurred at either cases’ home or work, so the choice of postcode samples is a clear candidate for improvement of the model. Possible alternatives to the current scheme could involve the forming of postcode samples based on replacing locations making a small contribution to the overall likelihood or using a likelihood based on a weighted sum of likelihoods for all possible infection locations with weights, for example depending of time spend at each location. However, the potential gains of such a change may not be sufficient to compensate for any weaknesses of the underlying model.

Population data is often broken down into nighttime, day term time and day non-term time populations. All calculations presented here were performed using day term time populations only. Clearly, this is a possible source of error. The most straightforward course of action regarding this is to repeat the whole calculation replacing the day term time populations with one of the other populations. However, there may be merit in using different populations types for different cases. The main difficulty with this is what action to take if there are multiple cases within a postcode with different assigned population types. One could take a weighted average of the populations involved and it could be argued that this should be done for single case postcodes in an attempt to allow for the fact that some of the population, such as daytime workers, will not be present and therefore not exposed to the disease for large parts of the average day. Whether any such adjustments of the populations used would be significant given the level of accuracy of the overall model is debatable.

The search for possible source locations was restricted to postcodes within the postal district with the highest occurrence of the disease per head of population. This has been done for computational ease rather than any scientific belief that this is in some sense optimal. The IMT’s search for potential sources was not restricted in this way. It is clearly possible to have a source which is just outside the postal district with the highest prevalence. In which case its true position would not be considered in the search. There may be merit in considering a search area defined using distance from the possible locations. For example considering all postcodes that are within a fixed distance of one or all of the possible infection locations where the fixed distance is chosen based on the maximum known distance between source and infection for the disease under consideration.

It should be noted that a major simplifying assumption is that wind blows equally in all directions over the duration of the release, this may not be realistic and during the period of interest a strong wind direction may have caused material to drift in a particular direction.

The underlying model used has a number of weakness such as no temporal dependence including within the dose dependency, no modelling of the survivability of *Legionella* bacteria within the aerosol, constant wind and not being disease specific. Some of these factors can at least in part been thought as being contained within the super-parameter, *α*. For example, the source size element within *α*, (see S1 Appendix), can be considered to have factors allowing for survivability and transfer from the original water source to the aerosol. Here the aim of the modelling is to estimate the location of the source and whether the source for example is large with low survivability or small with high survivability does not affect any conclusion about location. However, using a more complex model would almost certainly require more complex input data and this would defeat one of the primary objectives of the model. Whereas, a simple mechanistic assumption driven model allows for comparison with traditional epidemiological investigations [22] whilst being agnostic of the potential source.

The model is intended to supplement epidemiological and analytic tools currently used in outbreak investigation and control, (see references within Introduction for examples). The model only requires case infection locations. These are most likely to be home, work or visit addresses, information which is routinely gather by IMTs and can be easily and accurately communicated to whose responsible for running the model. A more sophisticated model would require correspondingly more data such as detailed travel histories or metrological data. These additional data may not be readability available in a timely fashion. Also, with increased complicity of model and data generally comes increased uncertainty of the results. Further, the lower computational cost of a simpler model allows greater exploration of the effects of data and parameter uncertainty and sensitivity.

The model outputs a most likely cell for the source location and a confidence region. This readily understood confidence region can easily be communicated either graphically or as a list of likely locations, back to the IMT for consideration alongside other evidence and results. The fact that the model requires no knowledge of possible sources means that novel or mis-registered potential sources can be highlighted by the analysis whereas they could not be by a method or model needing *a priori* knowledge of the potential sources.

## Supporting information

S1 Appendix Derivation of mean of infection poisson distribution. This appendix outlines the formulation of Eq (1) of the main text including a very brief description of the Briggs standard deviation terms used.
(PDF)
