# Supplementary material for: Dispersion of Legionella bacteria in atmosphere: A practical source location estimation method
Source: PLoS One. 2019 Nov 25;14(11):e0224144. doi: 10.1371/journal.pone.0224144 (PMC6876933; doi:10.1371/journal.pone.0224144)
Supplement: S1 Appendix — This appendix outlines the formulation of Eq (1) of the main text including a very brief description of the Briggs standard deviation terms used. (PDF) [file pone.0224144.s001.pdf]

A Derivation of Mean of Infection Poisson Distribution

A.1 Expected Number of Cases

Consider an instantaneous release of size Q from a point source at (X, Y, H), subjected to a constant wind with velocity $(U, V, 0)$. In this situation the 'classical' puff model with reflection from the ground may be derived, (see for example [1, 2] or Llewelyn [3]). Integrating over time the concentration field on the ground is then approximately given by

$$\Phi(x,y) \quad = \quad \frac{Q}{\pi W}\frac{e^{-H^2/2\sigma_z^2}}{\sigma_z}\frac{e^{-(Uy-UY+xV-XV)^2/2W^2\sigma^2}}{\sigma}$$

where $W = \sqrt{U^2+V^2}$ is the wind speed and σ^2 and σ_z^2 are the horizontal and vertical turbulence 'variances' or lengthscales. Along the plume centreline it is found that $\Phi(x,y) = Qe^{-H^2/2\sigma_z^2}/(\pi W\sigma_z\sigma)$ which is the dominant contribution for the given wind direction.

If it is assumed that a release is over a fixed time window, and consists of a large number T of separate independent individual releases each associated with independent wind directions, but that the wind speed in all such directions is the same, say $W = \omega$, then can integrate around all directions to find the concentration field to be

$$\Phi = 2QT\frac{e^{-H^2/2\sigma_z^2}}{\omega\sigma_z\sigma}.$$

The expected number of cases λ at a point will be the number of people $P(x,y)$ multiplied by the effective probability of infection. If the dose inhalation is modelled by a Poisson process then

$$\lambda(x,y) = P(x,y)\left[1-\exp\left(-b\phi\Phi\right)\right]$$

where b is some breathing rate and ϕ is the probability that an inhaled bacterium is retained in the lung. Noting that practically the retained infecting dose is small, otherwise would get very high attack ratios in particular regions of space, gives that

$$\lambda(x,y) \approx 2QTb\phi\frac{e^{-H^2/2\sigma_z^2}}{\omega\sigma_z\sigma}P(x,y). \tag{A.1}$$

Alternative options may be available to model variation in wind speed (i.e. if a prevailing wind direction over time or a wind rose was know) but operationally this is unlikely to be known early in the response.

A.2 Standard Deviation Terms

The standard deviations σ and σ_z are modelled by adopting the Briggs formalism, (see for example [4]), a standard formula derived from atmospheric dispersion literature, (other formulations for σ exist in literature but are not considered here). This gives that at a distance r from the source

$$\sigma = \frac{ar}{(1+br)^c},\ \sigma_z = \frac{a_z r}{(1+b_z r)^{c_z}}$$

where the coefficients a, b, c (and z subscript counterparts) vary by Pasquill stability class and by whether the environment being modelled is urban or rural but are predefined and given in Table A.1. Using this Equation (A.1) becomes

$$\lambda(x,y) = \alpha\frac{(1+br)^c(1+b_z r)^{c_z}}{r^2}\exp\left[-\frac{H^2(1+b_z r)^{2c_z}}{2a_z^2 r^2}\right]P(x,y)$$

where $\alpha = \frac{2b\phi QT}{\omega a_z a}$. This is Equation (1) of the paper.

Environment	Pasquill type	a	b (m^{-1})	c	a_z	b_z (m^{-1})	c_z
Open-country	A	0.22	0.0001	0.5	0.2	0	–
	B	0.16	0.0001	0.5	0.12	0	–
	C	0.11	0.0001	0.5	0.08	0.0002	0.5
	D	0.08	0.0001	0.5	0.06	0.0015	0.5
	E	0.06	0.0001	0.5	0.03	0.0003	1
	F	0.04	0.0001	0.5	0.016	0.0003	1
Urban	A–B	0.32	0.0004	0.5	0.24	0.001	-0.5
	C	0.22	0.0004	0.5	0.2	0	–
	D	0.16	0.0004	0.5	0.14	0.0003	0.5
	E–F	0.11	0.0004	0.5	0.08	0.00015	0.5

Table A.1: Summary of Briggs parameters for various stability and environmental conditions

References

[1] Judith Legrand, Joseph R. Egan, Ian M. Hall, Simon Cauchemez, Steve Leach, and Neil M. Ferguson. Estimating the location and spatial extent of a covert anthrax release. *PLoS Computational Biology*, 5(1):e1000356, 2009.

[2] John M. Stockie. The mathematics of atmospheric dispersion modeling. *SIAM Review*, 53(2):349–372, 2011.

[3] Richard P. Llewelyn. An analytical model for the transport, dispersion and elimination of air pollutants emitted from a point source. *Atmospheric Environment*, 17(2):249–256, 1983.

[4] Steven R. Hanna, Gary A. Briggs, and Rayford P. Hosker Jr. *Handbook of Atmospheric Diffusion*. U.S. Dept. of Energy, 1982.
